# Size Effects of Microplastics on Embryos and Observation of Toxicity Kinetics in Larvae of Grass Carp (*Ctenopharyngodon idella*)

**DOI:** 10.3390/toxics10020076

**Published:** 2022-02-07

**Authors:** Chaonan Zhang, Zhiheng Zuo, Qiujie Wang, Shaodan Wang, Liqun Lv, Jixing Zou

**Affiliations:** 1 Joint Laboratory of Guangdong Province and Hong Kong Region on Marine Bioresource Conservation and Exploitation, College of Marine Sciences, South China Agricultural University, Guangzhou 510642, China; zhangchaonan@stu.scau.edu.cn (C.Z.); zuozhiheng@stu.scau.edu.cn (Z.Z.); wongchaukit@stu.scau.edu.cn (Q.W.); 20201067007@stu.scau.edu.cn (S.W.); 2 Guangdong Laboratory for Lingnan Modern Agriculture, South China Agricultural University, Guangzhou 510642, China; 3 National Pathogen Collection Center for Aquatic Animals, Shanghai Ocean University, Shanghai 201306, China; lqlv@shou.edu.cn

**Keywords:** microplastic, grass carp, size, accumulation, re-consumption

## Abstract

Microplastics have caused great concern in recent years. However, few studies have compared the toxicity of different sizes of microplastics in fishes, especially commercial fishes, which are more related to human health. In the present study, we revealed the effects of varying sizes of microplastics on grass carp embryos and larvae using scanning electron microscopy (SEM) and fluorescence imaging. Embryos were exposed to 80 nm and 8 μm microplastics at concentrations of 5, 15, and 45 mg/L. Toxicity kinetics of various sizes of fluorescent microplastics were analyzed through microscopic observation in the larvae. Results found that nanoplastics could not penetrate the embryo’s chorionic membrane, instead they conglutinated or aggregated on the chorion. Our results are the first to explore the defense mechanisms of commercial fish embryos against microplastics. Larvae were prone to ingesting their own excrement, resulting in microplastic flocculants winding around their mouth. For the first time, it was found that excreted microplastics could be reconsumed by fish and reaccumulated in the oral cavity. Microplastics of a certain size (1 μm) could be accumulated in the nasal cavity. We speculate that the presence of a special groove structure in the nasal cavity of grass carp larvae may manage to seize the microplastics with a particular size. As far as we know, this is the first report of microplastics being found in the nasal passages of fish. Fluorescence images clearly recorded the toxicity kinetics of microplastics in herbivorous fish.

## 1. Introduction

The last five years have witnessed a rapid surge of published articles on microplastic pollution, which testifies to the great concern this pollutant has posed in recent years [1,2]. Although first raised as an issue by Thompson et al., 2004 [3], microplastics were first discovered in North America in the 1970s in the form of small spheres in plankton off the coast of New England [4]. Subsequently, other researchers also found that these tiny particles were not only in the aquatic environment [5,6,7], but also in soil [8,9], organisms [10,11,12], and even in the atmosphere [13,14]. According to the US National Oceanic and Atmospheric Administration (NOAA) in 2008, plastics smaller than 5 mm in size were identified as microplastics (MPs) [15]. With the development of cognition and technology, smaller microplastics were classified into nanoplastics (NPs). Although not clearly defined, particles within 100 nm in scale were commonly referred to as nanoplastics [16,17,18]. The 21st century has been called the age of plastics [19], largely because plastics are indispensable in contemporary life. Unfortunately, used plastics are not recycled or managed well, resulting in an increasing amount of waste getting discarded into the environment every year [20,21]. After physical, chemical, and biological degradation, plastics turn into microplastics or nanoplastics, which have become a threat to the ecological environment and human health [22,23]. People are now horrified by their huge numbers and extremely worried about the potential threat microplastics pose when they enter living organisms, because it means the plastics could threaten our health through the food chain, and even through drinking and simply breathing [24,25,26].

Many researchers have focused on the impact of microplastics on aquatic organisms, especially on algae [27,28,29] and shellfish [30,31], whereas relatively few studies have been conducted on fish [32,33]. In addition to the type, shape, concentration, and color of microplastics, particle size is one of the key factors influencing microplastics toxicological effects [34,35,36]. In general, the smaller particle size, the more toxic they are to organisms [16,17,18]. Specifically, on the one hand, microplastics with larger specific surface areas can adsorb more pollutants, resulting in enhanced toxicity. On the other hand, the smaller size of the microplastics, the longer they are retained in the body, increasing the risk of potential damage. For example, Ivleva et al. (2017) found rapid accumulation of <15 μm microplastics and concluded that smaller particles were of more concern than the larger ones [37]. Both 0.05 and 10 μm microplastics increased oxidative stress in marine copepod, but smaller microplastics raised more reactive oxygen species (ROS) [38]. The growth and reproduction of copepod showed a size-dependent decline after exposure to microplastics for 16 d [39]. These studies speculated that the effects of microplastics with different sizes on organisms are different, and toxicity usually increases with decreasing size. However, few studies compared the toxicity of varying sizes of microplastics in fish, especially commercial fish. Commercial fish refers to fish that can be bought in the market and cooked in the kitchen, and are more directly related to human health.

Compared to adult fishes, larvae are more sensitive to environmental stress [40,41]. Especially in its early stages, the pigment on the fish body surface is not fully formed, but the fish can feed and swim freely, making them ideal specimens to study dynamic distribution processes of microplastics in the body [42]. Fish eggs with lipophilic chorionic membranes could be potential surfaces for increased microplastic deposition and accumulation. Both periods (the larval and eggs) are critical for fish populations because of their high sensitivity to pollutants [43,44]. Batel et al. (2018) found that smaller and heavier microplastics (1–5 mm) accumulated in high numbers on the surface of zebrafish egg chorions [45]. Zhang et al. (2020) speculated that weak physical forces and/or electrostatic interactions operated between the chorion membrane and microplastics [46]. Fluorescence images of accumulation and egestion of microplastics in filter feeding tadpoles (*Xenopus tropicalis*) were concentration dependent [47]. The impacts of microplastics on embryo and larval fish can be directly reflected by fluorescence micrograph and SEM images. Our research group have focused on the differences of toxicity kinetics of microplastics in larvae with three feeding types and found that the effects of microplastics on fish were species-specific [42]. The results showed that the ingestion of microplastics in hybrid snakehead (carnivores) was lower than that in bighead carp (filter feeders) and mrigal (omnivores), while mrigal larvae were less effective to remove microplastics than bighead carp larvae. There is little research available on herbivorous fish [48], since this species is fewer, and samples are hard to obtain. However, grass carp (*Ctenopharyngodon idella*), as the typical representative of herbivorous fish, is a commercial fish with the largest amount of aquaculture in China [49,50].

In the present study, grass carp embryo and larvae were the model organisms, and different sizes of polystyrene microspheres were the exposure xenobiotics. Embryos at 12 h post fertilization (hpf) were exposed to 0.08 and 8 μm microplastics at various concentrations. In order to facilitate observation, green and red fluorescent microplastics were selected to visually reflect the dynamic distribution processes of microplastics in larvae. Toxicity kinetics of microplastics were analyzed through microscopic observation. This is the first study to investigate the accumulation, distribution, and egestion of microplastics in grass carp larvae. Therefore, our results aimed at bridging the gap on effects of microplastics in herbivorous fish.

## 2. Materials and Methods

### 2.1. Microplastics and Fish

We used microspheres with mean diameters of 0.08 and 8 μm (Dae Technology Co., Ltd., Tianjin, China) for the embryo toxicity assay, and fluorescent microspheres for larval exposure and elimination experiments. Green fluorescent polystyrene microspheres (excitation wavelength: 488 nm; emission wavelength: 518 nm) with mean diameters of 0.5 and 5 μm were purchased from Dae Technology Co., Ltd. (Tianjin, China). Orange fluorescent polystyrene microspheres (excitation wavelength: 540 nm; emission wavelength: 580 nm) with mean diameter of 1 μm were bought from the same company. Red fluorescent polystyrene microspheres (excitation wavelength: 620 nm; emission wavelength: 680 nm) with mean diameter of 5 μm were bought from Tianjin BaseLine ChromTech Research Centre (Tianjin, China). SEM figures of all kinds of microspheres are shown in Supplementary Figure S1.

The embryos of grass carp obtained from a stock farm in Qingyuan city, Guangdong Province, China, were packed in oxygenated bags and transferred to the lab immediately. They were then acclimatized in a 100 L glass tank prior to the exposure test. The dechlorinated circulating water conditions were as follows: water temperature 25.4 ± 1.3 °C, pH 7.0 ± 0.3, dissolved oxygen 6.5 ± 0.6 mg/L, and 14 h light/10 h dark photoperiod. The animals used in the present study were cultured and sacrificed following the terms of use of animals approved by the Animal Care and Use Committee of South China Agricultural University (identification code: 20210236; date of approval: 27 May 2021).

### 2.2. Embryo Toxicity Assay

The experimental embryos of grass carp were all in organogenesis stage (12 hpf). Microspheres with two sizes (0.08 and 8 μm) and at three concentrations (5, 15, and 45 mg/L) were used for the embryo toxicity assay. Each of the 15 embryos were assigned to glass Petri dishes with a diameter of 5 cm containing 5 mL test solution at random. There were two control groups that did not contain microplastics. The experiment was repeated three times. A total of 360 individuals and 24 glass Petri dishes were used. Embryo mortality was observed and recorded every two hours. The embryos were considered dead when they turned white.

### 2.3. SEM Analysis of Embryo

After 2, 4, 6, and 8 h exposure, embryos were collected and analyzed as described by [42,51], with slight modifications. The two sample preparation methods are as follows: (a) critical point drying: embryos were fixed in 4% paraformaldehyde for more than 24 h, rinsed thrice with 0.1 M phosphate-buffered saline (PBS, pH 7.4) for 15 min, and postfixed with 1% osmium tetroxide for 1.5 h at room temperature. Dehydration was carried out sequentially with ethanol concentrations of 30%, 50%, 70%, and 90% once for 10 min, followed by 100% ethanol twice for 10 min. After dehydration, samples were replaced with isopentyl acetate twice for 15 min, then dried in critical point desiccators (EP CPD300, Leica, Germany) overnight and stored at room temperature for SEM analysis; (b) freeze drying method: embryos were fixed in 3% glutaraldehyde for more than 24 h, and rinsed six times with 0.1 M PBS for 20 min. The dehydration procedure was similar to method (a), followed by replacement with tert-butanol twice for 20 min. After dehydration, embryos were dried in a vacuum freeze dryer (ES2030, Hitachi, Japan) and stored at room temperature for SEM analysis.

Before observation, samples were sputter-coated with an electrically conductive gold-palladium alloy in vacuum via a High Vacuum Sputter Coater (Leica EM ACE600, Germany). SEM images were taken with a Zeiss EVO MA 15 scanning electron microscope (Carl Zeiss AG, Jena, Germany) and FEI Verios 460 scanning electron microscope (Thermo Fisher Scientific, Waltham, MA, USA).

### 2.4. Exposure and Elimination Experiment of Larvae

The experimental larvae were hatched from normal fertilized eggs in clean water. We chose larvae that hatched after 24 h for the exposure and elimination experiment. They were exposed to 10 mg/L microplastics with diameters of 0.5 and 5 μm (green fluorescent microplastics) and 1 and 5 μm (red fluorescent microplastics), respectively, for four days. During the experiment, five samples from each group were taken out every 12 h and rinsed with clean water, and photographed under the fluorescence microscope (Nikon C-HGFI) equipped with a Nikon SMZ18 camera.

For the elimination experiment, the remanent larvae were transferred into 200 mL glass beakers containing clean water for four days. Each of the three samples were chosen every 12 h, rinsed with clean water, and photographed as described before.

## 3. Results

### 3.1. Effects of Microplastics on Embryos

There were no significant differences in the survival rates of grass carp embryos among all groups after 8 h exposure (Supplementary Figure S2). Even in a very high concentration of microplastics (45 mg/L), embryos could still hatch normally. There was no difference in morphology or fetal heart rate either.

### 3.2. Effects of Microplastics on Chorion Membranes

In order to maintain the stereoscopic morphology of the embryo, we used two sample preparation methods for SEM analysis. Unfortunately, the size of the fertilized eggs of grass carp was about 4 mm, and chorion membranes were shriveled or deformed to varying degrees after drying (Supplementary Figure S3) due to the technical difficulty.

**Figure 1:**
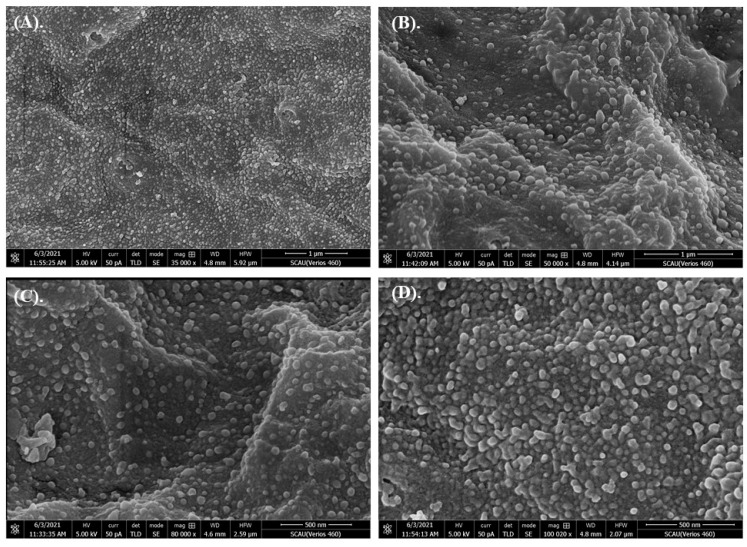
High-definition enlarged images of chorion membranes of grass carp. (**A**–**D**) show different parts of chorion membranes.

**Figure 2:**
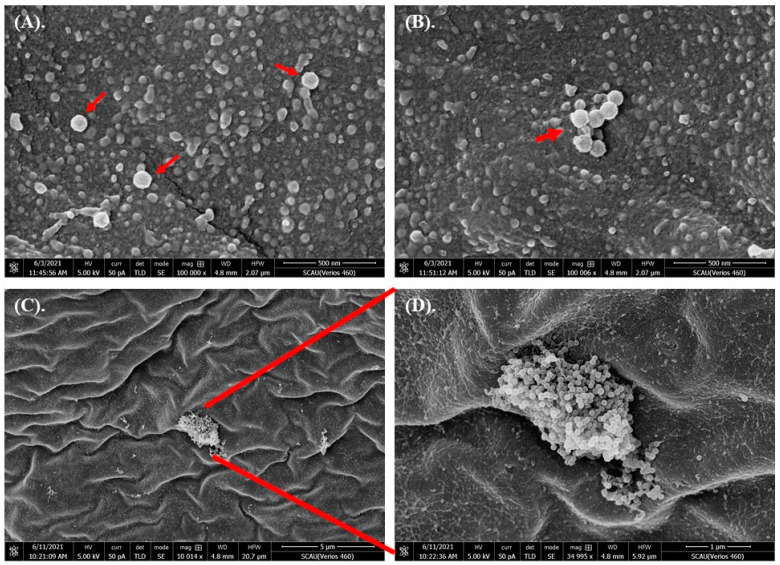
SEM images of the out-membrane surface of grass carp embryo after exposed to 80 nm microplastics. (**A**–**C**) show different status of microplastics on membranes. (**D**) is a larger version of (**C**).

**Figure 3:**
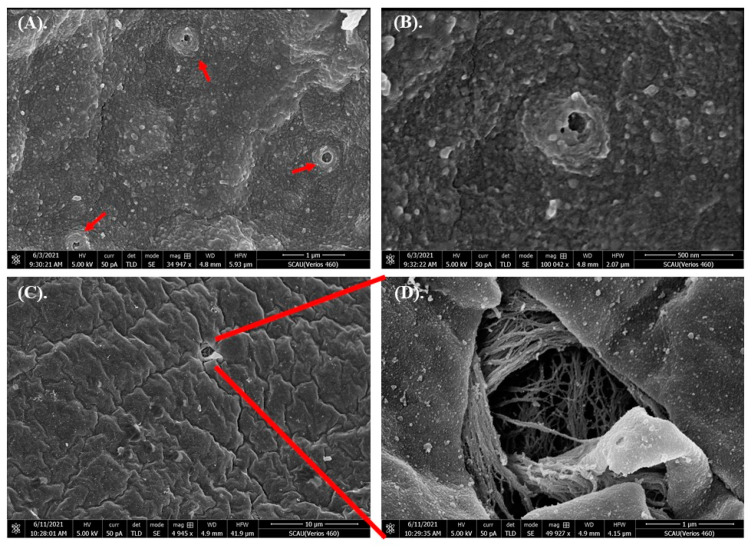
The pore structures of the out-membrane surface of grass carp embryo after exposed to microplastics. (**A**–**C**) show different pore structures. (**D**) is a larger version of (**C**).

High-definition enlarged images showed that the membrane surface was uneven, and there were many irregular protuberances (Figure 1). 80 nm microplastics were conglutinated or aggregated on the embryo chorion (Figure 2). The pore structures were observed in some embryos (Figure 3), but whether they were caused by microplastics was unclear. In critical point drying, the pores on the membrane surface appeared to be torn open to show a fibrous structure (Figure 3C,D). In addition, rod-shaped bacteria appeared and attached to some of the membrane surface (Supplementary Figure S4).

### 3.3. Uptake and Accumulation of Green Fluorescent Microspheres in Grass Carp Larvae

**Figure 4:**
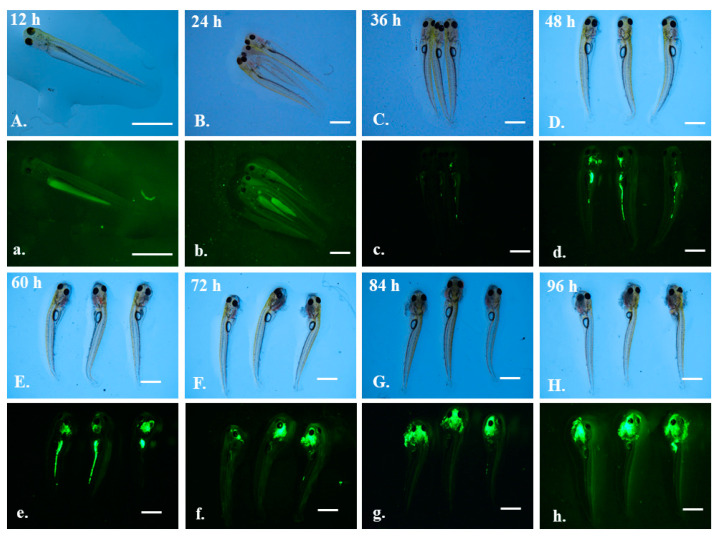
The larvae of grass carp after exposure to 5 μm green fluorescent microplastics. Photographs were taken under a brightfield microscope (capital letters **A**–**H**) and green fluorescent microscope (lowercase letters **a**–**h**). Observation time was labeled in the figure. Scale bar = 2 mm.

Grass carp larvae (about 9 mm in length) were observed to the microplastics exposure experiment for four days. During the first 24 h of exposure, green autofluorescence was observed in the thoracic cavity of the larvae, both in the control (Supplementary Figure S5a,b) and exposed groups (Figure 4a,b). After three days of exposure, autofluorescence in the larvae faded, leaving remnant fluorescence in the yolk sac. Photographs of the control group under fluorescent lenses are shown in Supplementary Figure S5.

In the exposed group, 5 μm microplastics gradually accumulated in the intestines of the larval grass carps from 36 h to 60 h (Figure 4c–e). However, from 72 h to 96 h, there was no fluorescent signal in the intestines, and all the microplastics accumulated in the oral cavity (Figure 4f–h). Under a brightfield microscope, obvious flocculation could be observed around the oral cavity (Figure 4F–H).

In the exposed 72–96 h of 0.5 μm microplastics, the fluorescent particles in some of the intestinal tracts were not removed (Supplementary Figure S6f–h), while most of the microplastics accumulated in the oral cavity. The accumulation of 0.5 μm microplastics during 36–60 h was similar with that of 5 μm microplastics (Supplementary Figure S6c–e).

### 3.4. Uptake and Accumulation of Red Fluorescent Microspheres in Grass Carp Larvae

**Figure 5:**
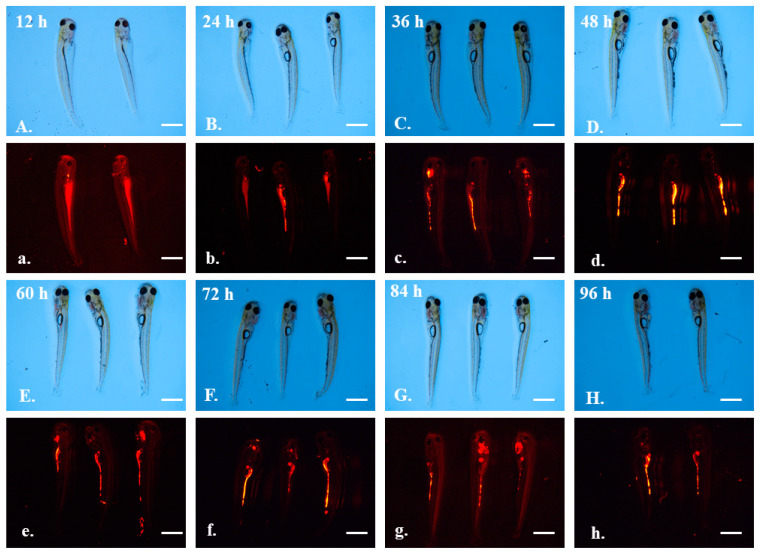
The larvae of grass carp after exposure to 5 μm red fluorescent microplastics. Photographs were taken under a brightfield microscope (capital letters **A**–**H**) and red fluorescent microscope (lowercase letters **a**–**h**). Observation time was labeled in the figure. Scale bar = 2 mm.

There was no red fluorescence in grass carp larvae of the control group (Supplementary Figure S7). However, grass carp larvae after exposure to 5 μm red fluorescent microplastics showed red autofluorescence in the thoracic cavity at 12–24 h (Figure 5a,b). After 36 h of exposure, red fluorescence appeared in a strip shape, indicating that the 5 μm red fluorescent microplastic had entered the intestines of the larval grass carp (Figure 5c–h). Autofluorescence in the thorax of grass carp was band-shaped. Unlike 5 μm green fluorescent microplastics, 5 μm red fluorescent microplastics accumulated in the intestines during exposure.

**Figure 6:**
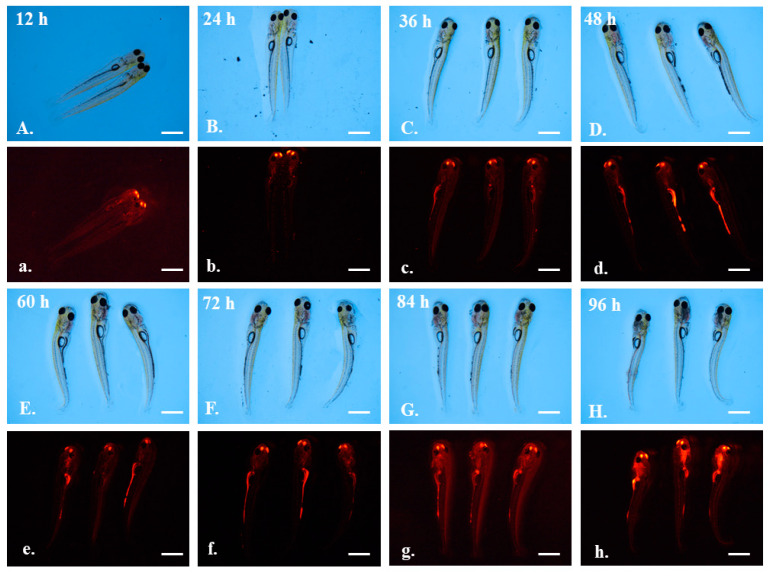
The larvae of grass carp after exposure to 1 μm orange fluorescent microplastics. Photographs were taken under a brightfield microscope (capital letters **A**–**H**) and red fluorescent microscope (lowercase letters **a**–**h**). Observation time was labeled in the figure. Scale bar = 2 mm.

The accumulation sites of 1 μm fluorescent microplastics were different from those of 5 μm fluorescent microplastics. At 24 h after exposure, red fluorescent signals appeared at the nose of the larval grass carp (Figure 6a,b). After 36 h of exposure, 1 μm microplastics gradually entered the intestines, but the red fluorescent signal in the nose was still not eliminated (Figure 6c–h). Notably, after 96 h, microplastics seemed to be more concentrated around the oral cavity (Figure 6h). Under a brightfield microscope, obvious floccules could be observed (Figure 6H).

### 3.5. Elimination of Green Fluorescent Microspheres in Grass Carp Larvae

The elimination test also lasted for four days. No fluorescent microplastics were found in the intestines of grass carps in the control group (Supplementary Figure S8). As shown in Supplementary Figure S9, after 4 days of exposure to 5 μm green fluorescent microplastics, floccules and fluorescent substances around the oral cavity of the larval grass carps did not disappear during four days of the elimination test, while the larvae could swim normally. The cleaning situation was similar for larvae exposed to 0.5 μm green fluorescent microplastics (Supplementary Figure S10). It is worth noting that grass carps in the control group did not have flocculent entanglement near their mouths.

### 3.6. Elimination of Red Fluorescent Microspheres in Grass Carp Larvae

No fluorescent microplastics were found in the intestines of grass carps in the control group (Supplementary Figure S11). We observed that 5 μm red fluorescent microplastics accumulated in the intestines of grass carps during exposure. Over the elimination course of 48 h, microplastics were gradually removed from the intestines (Supplementary Figure S12a–d). During the 60–96 h of elimination, red fluorescence mainly concentrated in the oral cavity of grass carps, and floccules also appeared at this time (Supplementary Figure S12e–h).

**Figure 7:**
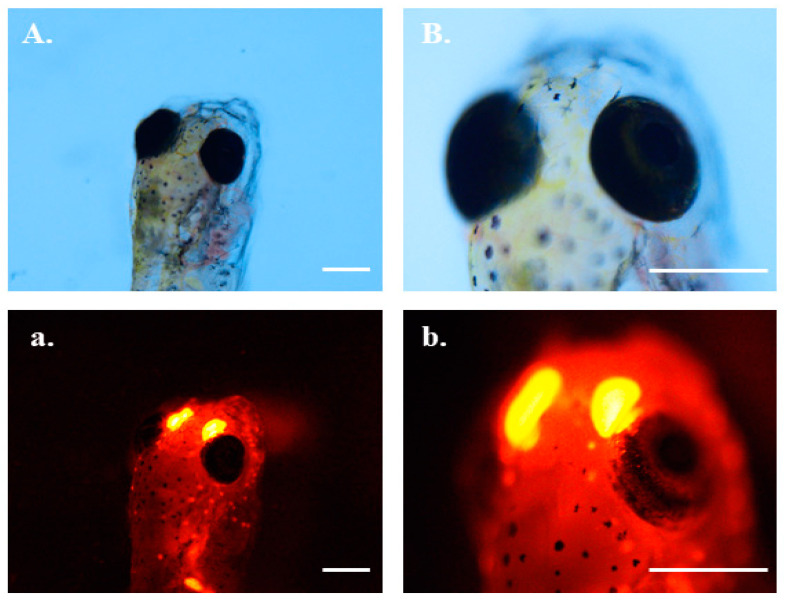
The larvae of grass carp after exposure to 1 μm red fluorescent microplastics. Photographs were taken under a brightfield microscope (capital letters **A**,**B**) and red fluorescent microscope (lowercase letters **a**,**b**). **B/b** is a larger version of **A/a**. Scale bar = 0.5 mm.

Orange fluorescent microplastics with 1 μm size in the grass carp intestines were removed from the body at the early stage of the elimination experiment (within 12 h). However, the fluorescence in the nose always existed (Supplementary Figure S13). The close-up is shown in Figure 7. From the images of the larvae, we could not determine whether the fluorescence was in the nasal region. Compared with the appearance of adult grass carp (Supplementary Figure S14), we found that the nasal cavity of grass carp was very obvious.

## 4. Discussion

### 4.1. Effects of Microplastics on Embryos

We studied the effects of microplastics of different sizes and varying concentrations on grass carp embryos. Results showed that embryos at 12 hpf were not affected by microplastics with nano size or high concentrations. SEM photos showed that microplastics centered and aggregated on the embryo chorion, but couldn’t penetrate into the interior. Fertilization and development of fish eggs are in vitro. Nutrients needed for the development of the embryo come from the yolk, and there is little need to obtain nutrients or excrete waste from outside the embryo. During the development of the embryo, the dense chorionic membrane structure is helpful for protection, since the fish eggs have to face various environmental stresses. However, the function of irregular protuberances on the membrane surface (Figure 1) was unclear, and adverse effects caused by the tiny particles on chorion was unmeasurable. Our results were similar with [46], in which they also found that microplastics could be adsorbed on the outer membrane surface making the membrane layer irregular in zebrafish embryos after being exposed to 10 μm microplastics at 10 mg/L for 48 h. They deduced that there were weak physical forces and/or electrostatic interactions between the chorion membrane and microplastics. Another report showed that silver nanoparticles with an average diameter of 11.6 nm were passively diffused into zebrafish embryos through chorion pore canals [52]. However, most research results supported the conclusion that no overt embryotoxicity occurred when nanoparticles aggregated on the chorion of embryos [53].

Fish eggs can be divided into adhesive, pelagic, demersal, and floating eggs according to their specific gravity and viscosity. The zygotes of zebrafish, a model organism commonly used in the laboratory, are demersal eggs, which are characterized with a larger density than water and a smaller yolk gap [46]. However, the zygotes of grass carps used in this experiment are floating eggs, which are characterized by water absorption and expansion, large perivitelline space, and suspension in the water layer [54]. The differences in the surface chorionic membrane of various types of fish eggs might lead to the discrepancy in conglutination of microplastics, which have not been studied thoroughly. This could be of significant concern, and it is important to address the effects with individual differences.

### 4.2. Effects of Microplastics of Different Sizes on Fish

The effects of 5 μm microplastics with green and red fluorescence exposure results were not the same, which suggested the importance in the selection of microplastic materials. This is likely because different materials would obtain different experimental results. Even when different groups of researchers use microplastics of the same size as the material, cross-sectional comparisons should be treated with caution. Fluorescent dye-labeled microplastics bring convenience to observation, but also create a certain confusion. Catarino et al. (2019) found that manufactured fluorescent microplastics leached their fluorophores, and fluorophores possibly accumulated in the zebrafish gut, rather than the microplastics themselves [55]. By carefully comparing our experimental results with those of Catarino et al. (2019), we confirmed that what entered the grass carp guts were fluorescent microplastics, rather than fluorophores. The biggest difference was whether they were distributed in bands or strips in the body. However, although it was confirmed that they were the same particle size of 5 μm, the difference of toxicity kinetics in red and green fluorescent microplastics during the exposure experiment could not be accounted for. Commercial microplastic pellets, especially those with fluorescence, need to be carefully selected and considered.

The green fluorescent microplastics sized 0.5 and 5 μm showed no size-dependent effects. They both accumulated mainly in the digestive and oral tracts of grass carp larvae via oral ingestion regardless of exposure and depuration time. In general, small particles led to prolonged retention time and high bioavailability. A number of past results indicated that uptake of microplastics in organisms significantly depended on particle size. For example, Lu et al. (2016) found both 5 and 20 μm microplastics in the intestines and gills of adult zebrafish, while only the smaller-sized microplastics in the liver [56]. In addition, although no significant differences between histopathological changes were observed in the tissues for fish exposure to the 70 nm and 5 μm microplastics, larger-sized microplastics induced increased activities of superoxide dismutase (SOD) and catalase (CAT). Yang et al. (2020) found that 70 nm microplastics could enter the epidermis more easily than 5 μm microplastics in goldfish larvae, leading to muscle mesenchymal cell damage and nerve fiber atrophy [57]. The size-dependence effects of 0.05, 0.5 and 6 μm microplastics on rotifers were observed, such as reduction of growth rate, lifespan, and fecundity [39]. The size range of microplastics causing differences of biological effects is species-specific, which may be closely related to the organism’s own tissue structure. Future research should focus on the interaction of microplastic size and the research object.

Interestingly, 1 μm orange fluorescent microplastics could accumulate in the nasal cavity of grass carp larvae, and could not be removed once they entered. We suspect that there is a special groove structure about 1 μm in the nasal cavity of grass carp larvae which manage to seize the microplastics with the particular size. As far as we know, this is the first report of microplastics being found in the nasal passages of fish. Recently, a study reported the accumulation of 23 nm microplastics in the brain of juvenile grass carp, which could cause multiple adverse effects, including impaired growth/development, behavioral changes, and anti-predatory defensive response associated with oxidative stress [58]. Another study found that microplastics were accumulated in gills close to blood vessels, indicating the respiratory system as one of the main egestion ways for microplastics in fish [59,60]. Microplastics with a diameter of 25 and 50 nm also accumulated in the eye, which could either be from outer epidermal or internal biodistribution through the intestinal epidermis [61]. The tissue specificity of microplastic accumulation in organisms and the resulting potential harm need to be studied further.

### 4.3. Excretion and Re-Consumption of Microplastics

In the 96 h of exposure, 5 μm red fluorescent microplastics accumulated in the digestive tract of grass carp larvae, and fluorescence intensity decreased during the elimination experiment. However, the green fluorescent microplastics, whether 0.5 or 5 μm in size, were excreted after 72 h exposure. The gut residence time of microplastics ingested by the fish seemed to be related to the fluorescent dye, independent of the size. But the retention time in rotifers likely correlated with the size of the microplastic [39]. The residence time of microplastics in organisms may depend on the gut space of organisms and the type, shape, size and concentration of the materials. The slow excretion of plastics might damage or block the digestive tract, thus affecting food consumption and the energy acquirement for vital functions. Moreover, longer retention times might prolong the negative effects. Most laboratory toxicology experiments use regular, smooth microspheres as experimental materials, which may have different residence times for experimental materials and field samples (such as fibers or fragments). The residence time of microplastics in fish and their effects are, however, still beyond our knowledge.

There was still strong fluorescent during depuration period, indicating that grass carp larvae could re-accumulate feces containing microplastics in the oral cavity. For the first time, it was found that excreted microplastics could be reconsumed by fish and reaccumulated in the oral cavity. We suspect that the mechanism of why the re-accumulated microplastics remained in the oral cavity is related to the mouth structure and fecal properties of grass carp larvae. The process of consuming-excreting-reconsuming microplastics may increase the potential for bioaccumulation. Such a process of reconsuming was not observed in the previous toxicity kinetics of carnivorous, omnivorous, and filter-feeding larvae [42]. Although most commercial freshwater fishes in the larval stage are planktivorous, the processes of uptake, accumulation, and elimination of microplastics are species-specific. Studies have shown that feces excreted by organisms after microplastics exposure carried microplastics, and changed the sedimentation rate, which was one of the major pathways for vertical translocation. Cole et al. (2016) hypothesized a mechanism in which floating plastics were transported out of surface water through a combination of microplastics and fecal pellets [62]. They found that the sinking rate of fecal pellets incorporated within microplastics decreased by 2.25-fold because of the reduction in density. However, another study pointed out that excreted polyethylene microplastics coated by intestinal liquids resulted in aggregation and sinking [36]. More studies are needed to further explain the deposition and transportation mechanisms of microplastics.

## 5. Conclusions

This study aimed to reveal the effects of varying microplastic particle sizes on grass carp embryos and larvae from the perspective of SEM and fluorescence imaging. The results showed that nanoplastics could not penetrate the chorionic membrane of the embryos, but could conglutinate and aggregate on the chorion. A high concentration of microplastics exposure did not affect the development of embryos during organ formation. Toxicity kinetics from green and red fluorescence microplastics with the same particle size (5 μm) exposure were unexpectedly different. Feces containing microplastics reaccumulated into the oral cavity. Green fluorescent microplastics of 0.5 and 5 μm showed no size-dependent effects. Microplastics of 1 μm accumulated in the nasal cavity. Further studies should pay more attention to the choice of microplastics as the materials and the fish as the model organisms.

## Data Availability

The data presented in this study are available in Section 4 and supplementary material.

## Supplementary Materials

The following supporting information can be downloaded at: https://www.mdpi.com/article/10.3390/toxics10020076/s1, Figure S1: SEM figures of all kinds of polystyrene microspheres: 5 μm green fluorescent microplastics (A); 0.5 μm green fluorescent microplastics (B); 5 μm red fluorescent microplastics (C); 1 μm orange fluorescent microplastics (D). Figure S2: The survival rates of grass carp embryos among all groups when exposure to 8 μm (A) and 80 nm (B) microspheres. Figure S3: Chorion membranes of grass carp after drying. The size of microplastics and exposure time are shown in the figure. Figure S4: Rod-shaped bacteria were attached to some of the membrane surface. Figure S5: The control group in brightfield microscope (capital letters) and green fluorescent microscope (lowercase letters). Observation time was labeled in the figure. Scale bar = 2 mm. Figure S6: The larvae of grass carp after exposure to 0.5 μm green fluorescent microplastics. Photographs were taken under a brightfield microscope (capital letters) and green fluorescent microscope (lowercase letters). Observation time was labeled in the figure. Scale bar = 2 mm. Figure S7: The control group in brightfield microscope (capital letters) and red fluorescent microscope (lowercase letters). Observation time was labeled in the figure. Scale bar = 2 mm. Figure S8: The control group during elimination test in brightfield microscope (capital letters) and green fluorescent microscope (lowercase letters). Observation time was labeled in the figure. Scale bar = 2 mm. Figure S9: The larvae of grass carp after depurating from 5 μm green fluorescent microplastics. Photographs were taken under a brightfield microscope (capital letters) and green fluorescent microscope (lowercase letters). Observation time was labeled in the figure. Scale bar = 2 mm. Figure S10: The larvae of grass carp after depurating from 0.5 μm green fluorescent microplastics. Photographs were taken under a brightfield microscope (capital letters) and green fluorescent microscope (lowercase letters). Observation time was labeled in the figure. Scale bar = 2 mm. Figure S11: The control group during elimination test in brightfield microscope (capital letters) and red fluorescent microscope (lowercase letters). Observation time was labeled in the figure. Scale bar = 2 mm. Figure S12: The larvae of grass carp after depurating from 5 μm red fluorescent microplastics. Photographs were taken under a brightfield microscope (capital letters) and red fluorescent microscope (lowercase letters). Observation time was labeled in the figure. Scale bar = 2 mm. Figure S13: The larvae of grass carp after depurating from 1 μm red fluorescent microplastics. Photographs were taken under a brightfield microscope (capital letters) and red fluorescent microscope (lowercase letters). Observation time was labeled in the figure. Scale bar = 2 mm. Figure S14: The appearance of adult grass carp. The area marked in the red box is the nasal cavity.
