# Supplementary material for: Size Effects of Microplastics on Embryos and Observation of Toxicity Kinetics in Larvae of Grass Carp (Ctenopharyngodon idella)
Source: Toxics. 2022 Feb 7;10(2):76. doi: 10.3390/toxics10020076 (PMC8877553; doi:10.3390/toxics10020076)
Supplement: Supplementary file 1 [file toxics-10-00076-s001.zip › toxics-1543766-supplementary.pdf]

toxics

MDPI

Supplementary Materials: Size Effects of Microplastics on Embryos and Observation of Toxicity Kinetics in Larvae of Grass Carp (*Ctenopharyngodon idella*)

Chaonan Zhang, Zhiheng Zuo, Qiujie Wang, Shaodan Wang, Liqun Lv and Jixing Zou

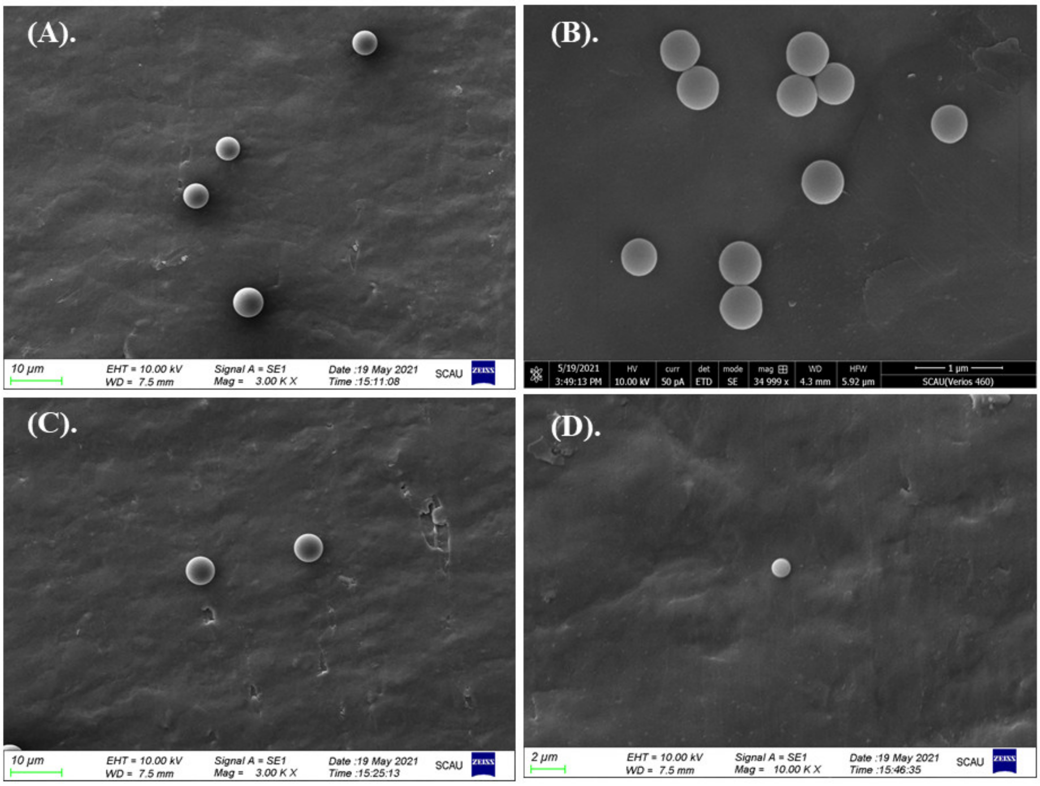

Figure S1. SEM figures of all kinds of polystyrene microspheres: 5 μm green fluorescent microplastics (**A**); 0.5 μm green fluorescent microplastics (**B**); 5 μm red fluorescent microplastics (**C**); 1 μm orange fluorescent microplastics (**D**).

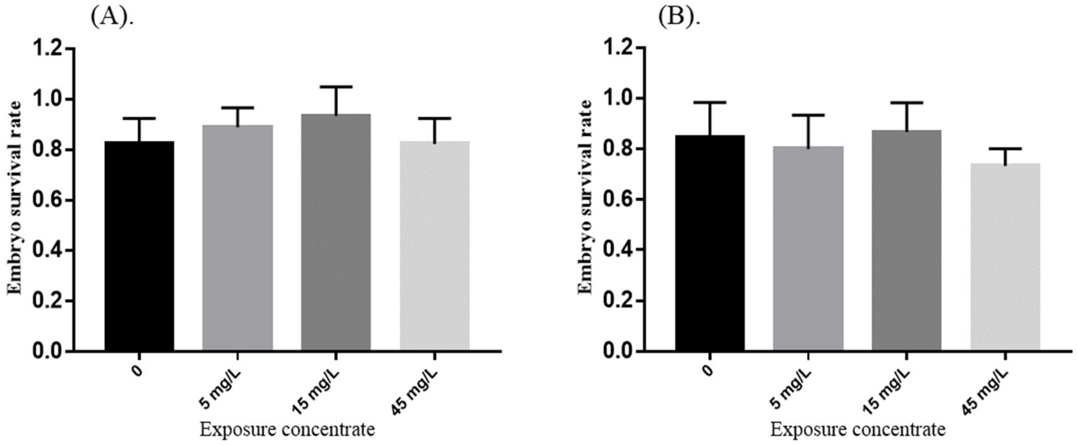

Figure S2. The survival rates of grass carp embryos among all groups when exposure to 8 μm (**A**) and 80 nm (**B**) microspheres.

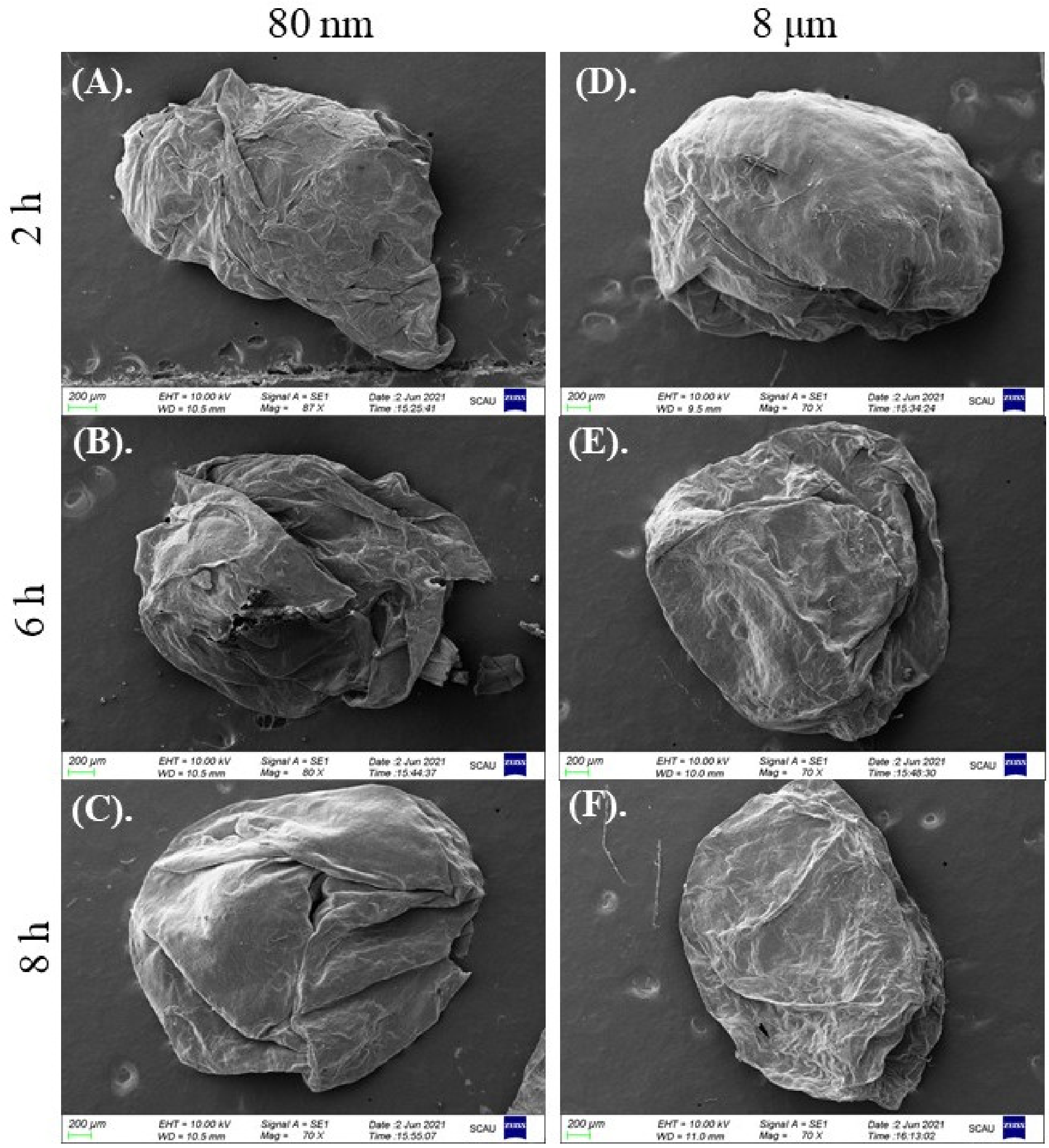

Figure S3. Chorion membranes of grass carp after drying. The size of microplastics and exposure time are shown in the figure.

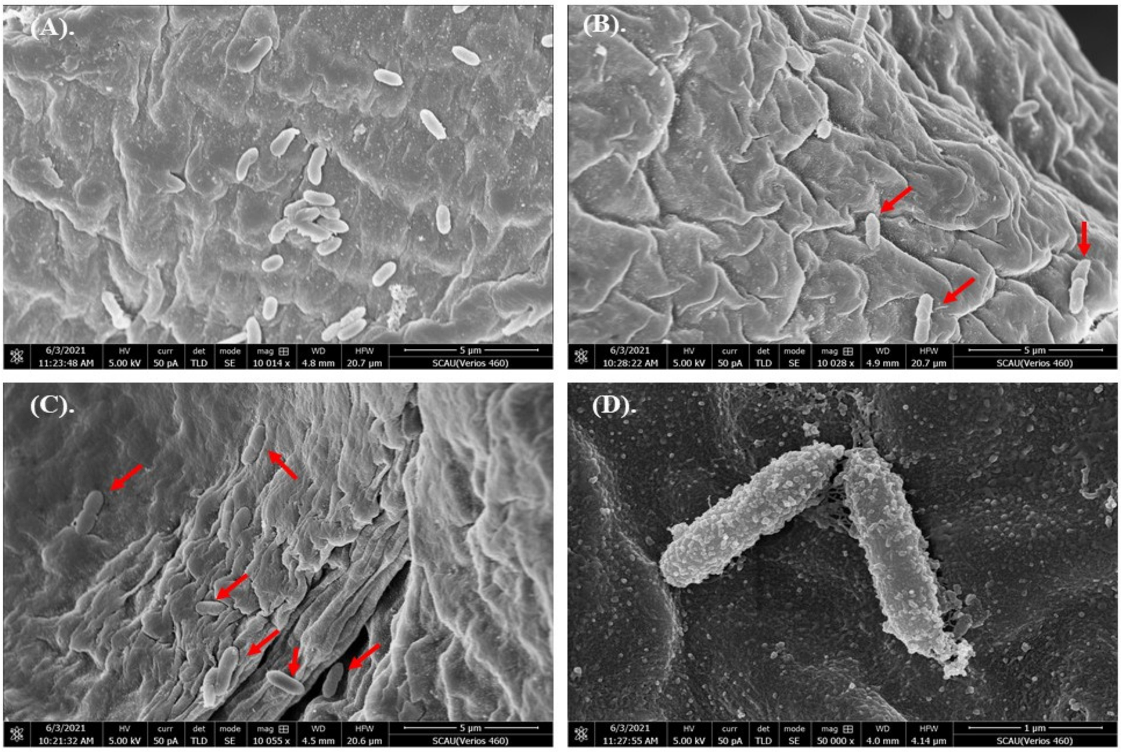

Figure S4. Rod-shaped bacteria were attached to some of the membrane surface. A-D show different forms of bacteria.

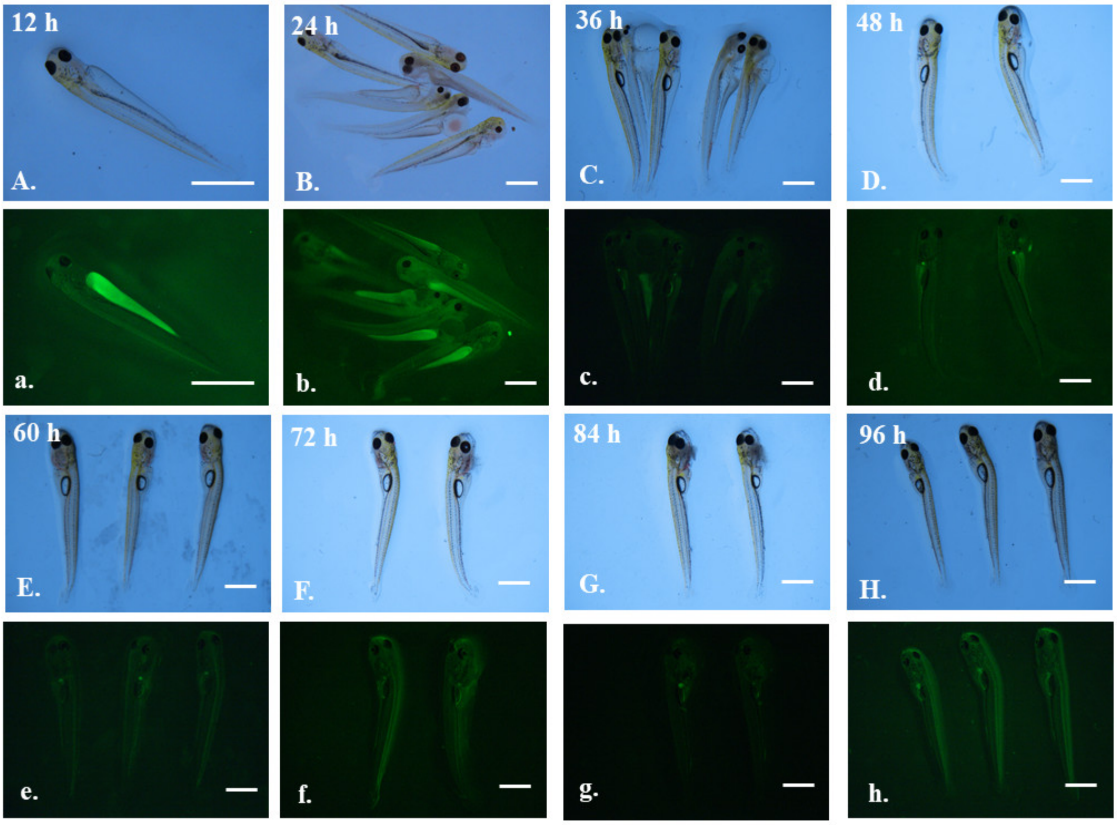

Figure S5. The control group in brightfield microscope (capital letters **A**–**H**) and green fluorescent microscope (lowercase letters **a**–**h**). Observation time was labeled in the figure. Scale bar = 2 mm.

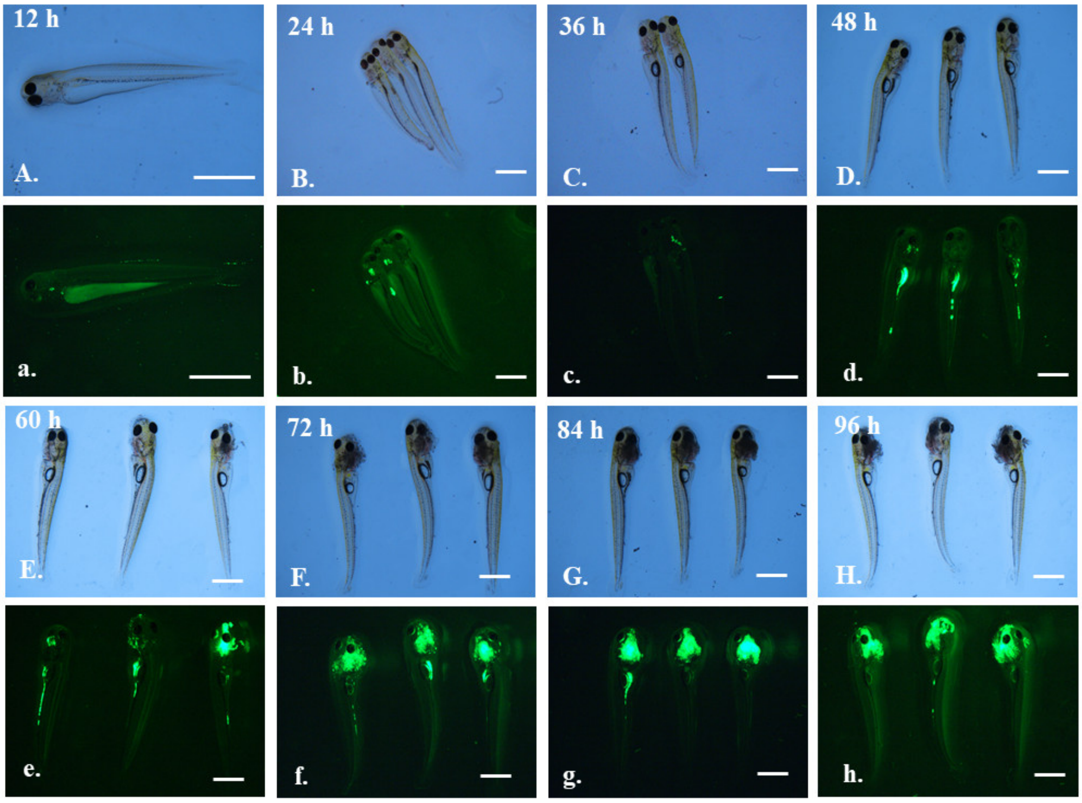

Figure S6. The larvae of grass carp after exposure to 0.5 μm green fluorescent microplastics. Photographs were taken under a brightfield microscope (capital letters **A**–**H**) and green fluorescent microscope (lowercase letters **a**–**h**). Observation time was labeled in the figure. Scale bar = 2 mm.

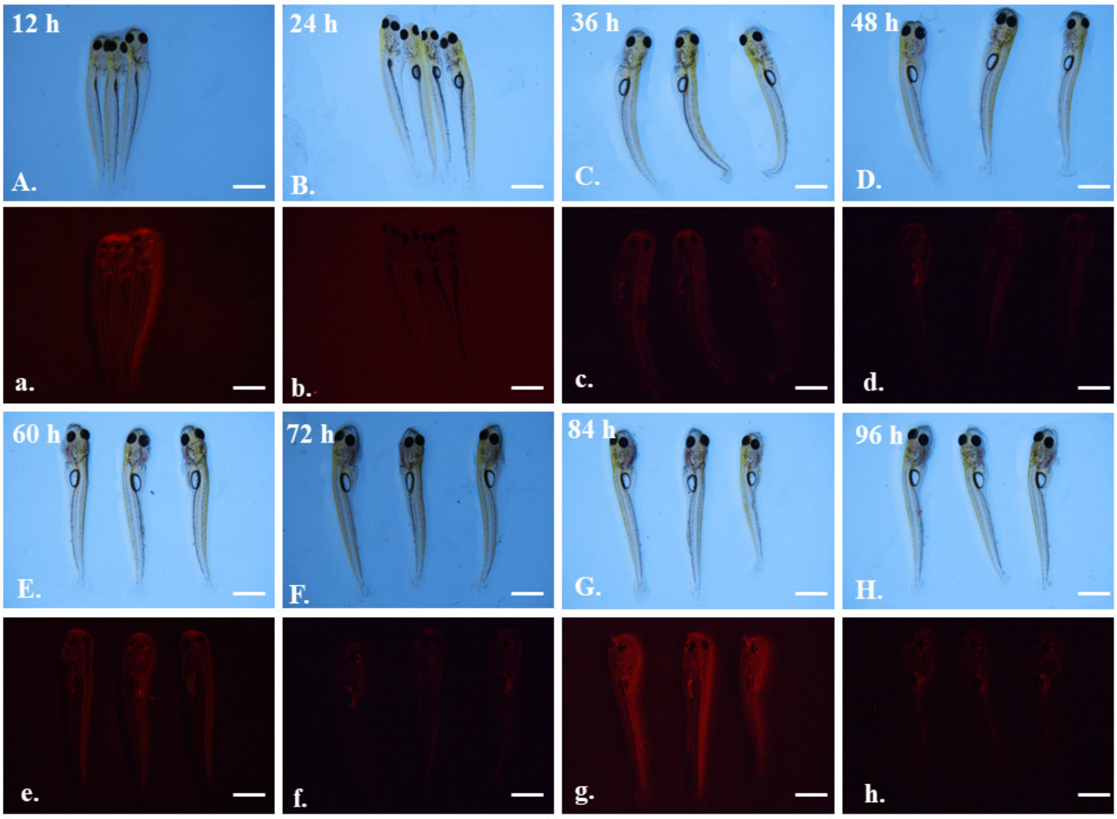

Figure S7. The control group in brightfield microscope (capital letters **A**–**H**) and red fluorescent microscope (lowercase letters **a**–**h**). Observation time was labeled in the figure. Scale bar = 2 mm.

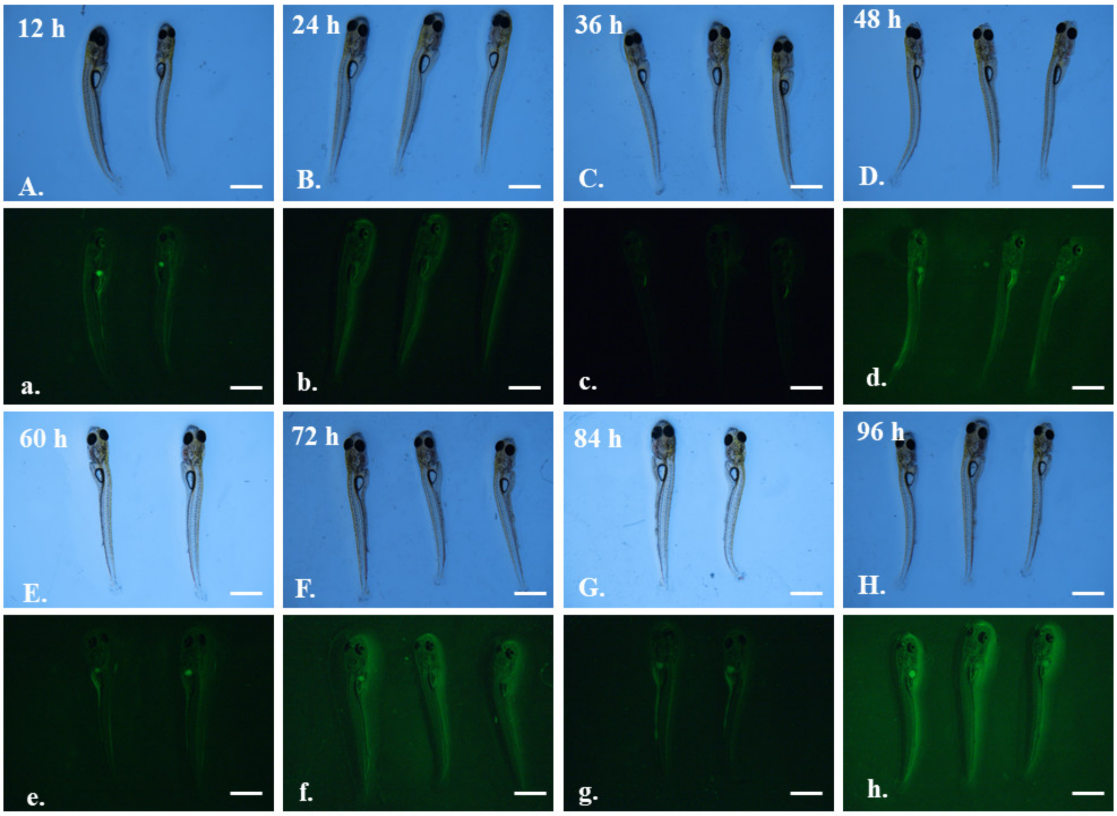

Figure S8. The control group during elimination test in brightfield microscope (capital letters **A**–**H**) and green fluorescent microscope (lowercase letters **a**–**h**). Observation time was labeled in the figure. Scale bar = 2 mm.

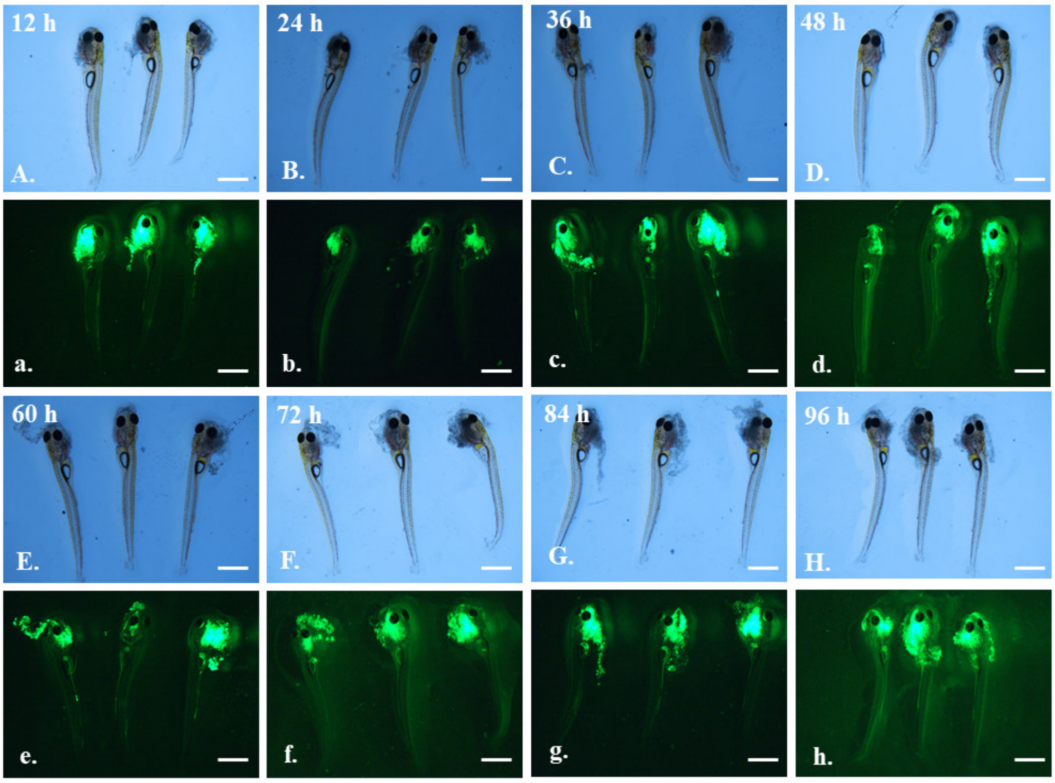

Figure S9. The larvae of grass carp after depurating from 5 μm green fluorescent microplastics. Photographs were taken under a brightfield microscope (capital letters **A**–**H**) and green fluorescent microscope (lowercase letters **a**–**h**). Observation time was labeled in the figure. Scale bar = 2 mm.

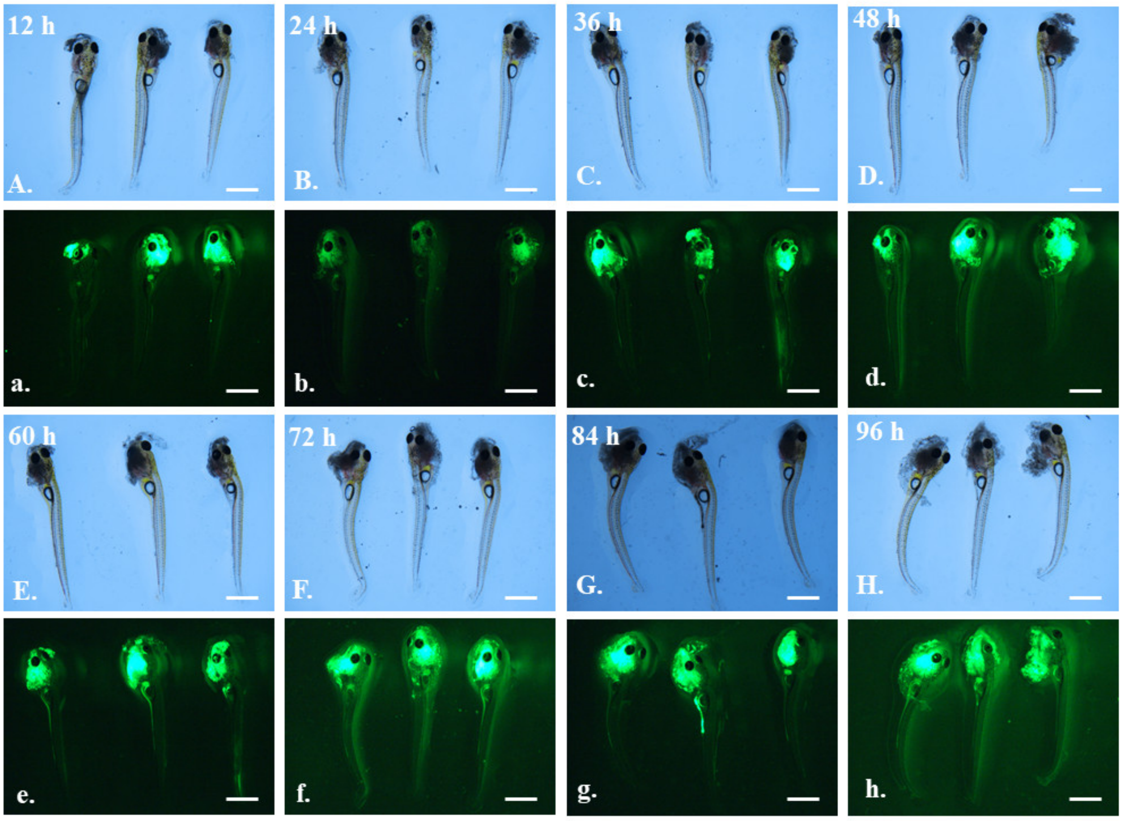

Figure S10. The larvae of grass carp after depurating from 0.5 μm green fluorescent microplastics. Photographs were taken under a brightfield microscope (capital letters **A**–**H**) and green fluorescent microscope (lowercase letters **a**–**h**). Observation time was labeled in the figure. Scale bar = 2 mm.

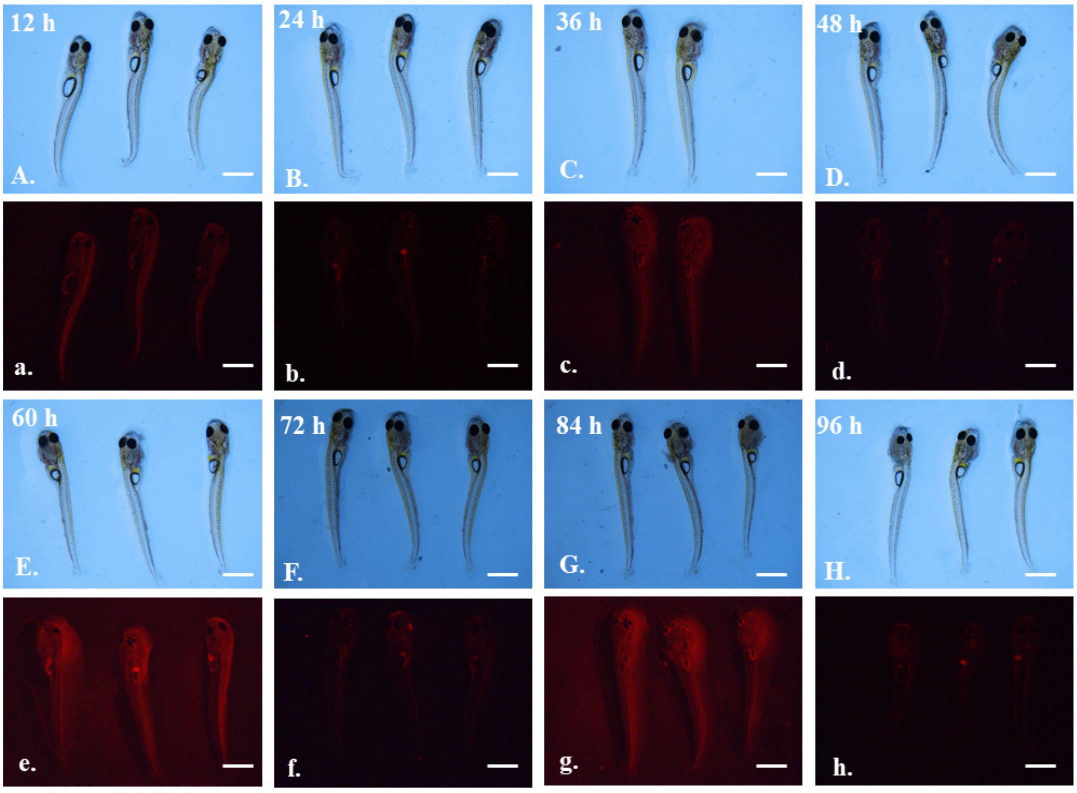

Figure S11. The control group during elimination test in brightfield microscope (capital letters **A**–**H**) and red fluorescent microscope (lowercase letters **a**–**h**). Observation time was labeled in the figure. Scale bar = 2 mm.

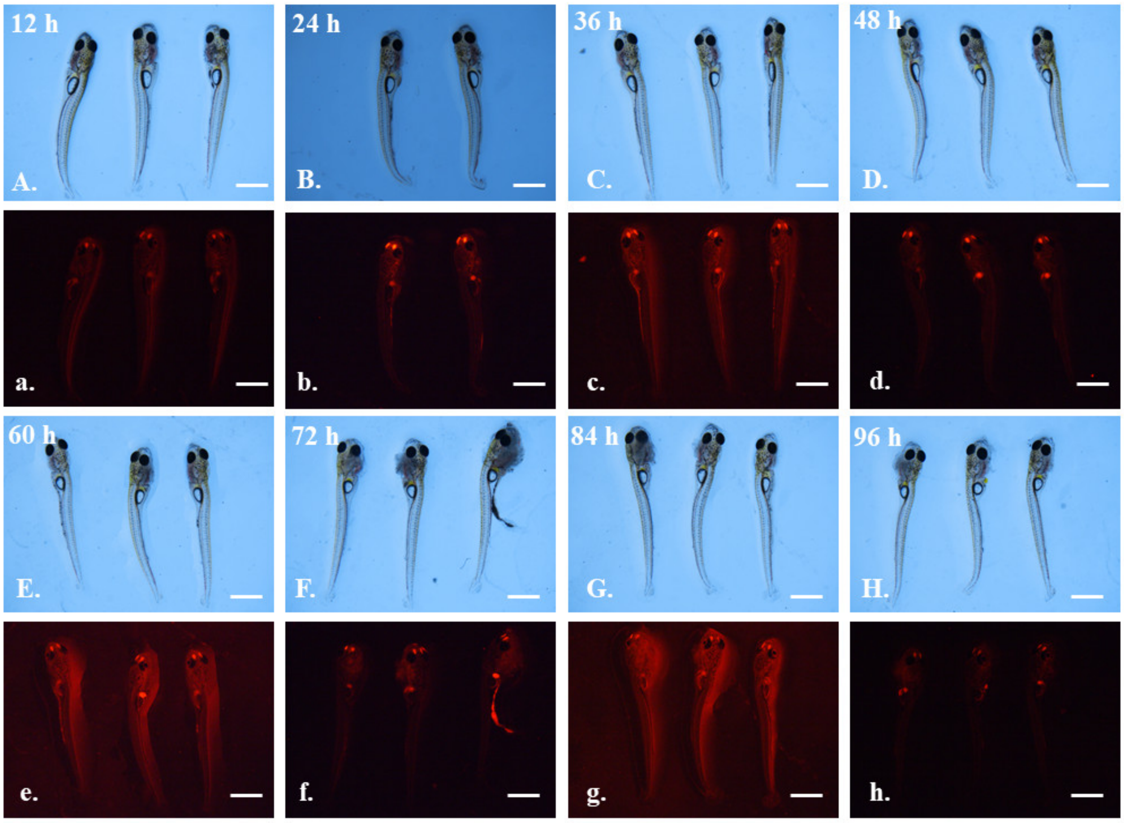

Figure S12. The larvae of grass carp after depurating from 1 μm red fluorescent microplastics. Photographs were taken under a brightfield microscope (capital letters **A**–**H**) and red fluorescent microscope (lowercase letters **a**–**h**). Observation time was labeled in the figure. Scale bar = 2 mm.

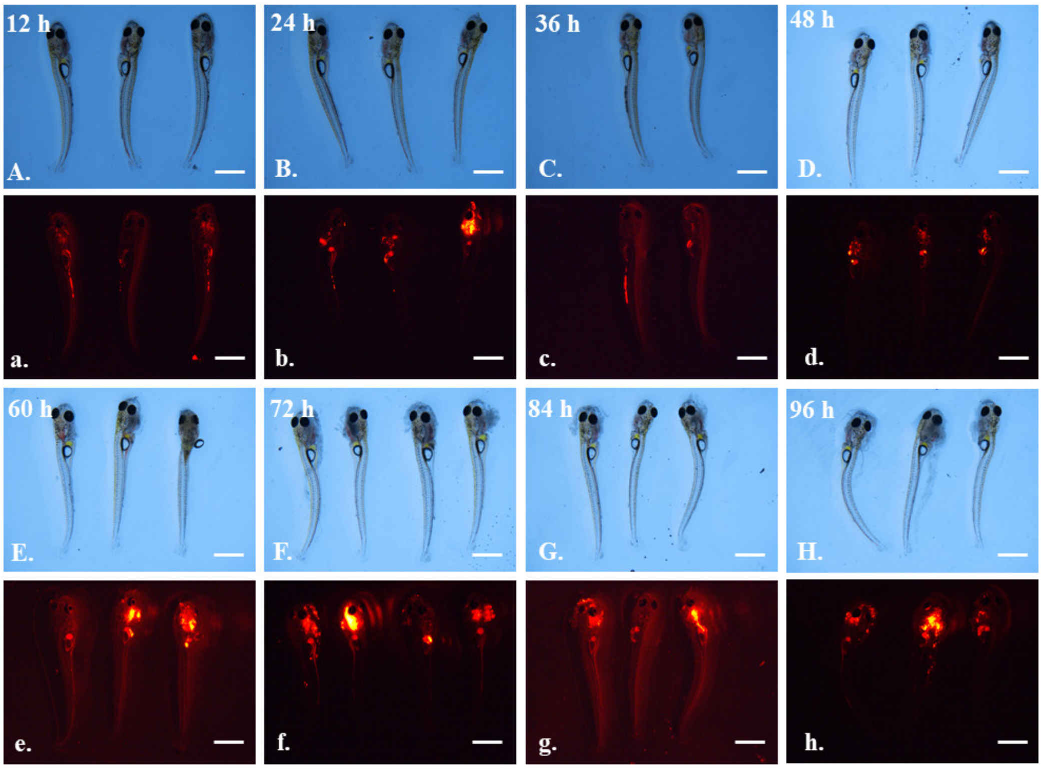

Figure S13. The larvae of grass carp after depurating from 5 μm red fluorescent microplastics. Photographs were taken under a brightfield microscope (capital letters **A**–**H**) and red fluorescent microscope (lowercase letters **a**–**h**). Observation time was labeled in the figure. Scale bar = 2 mm.

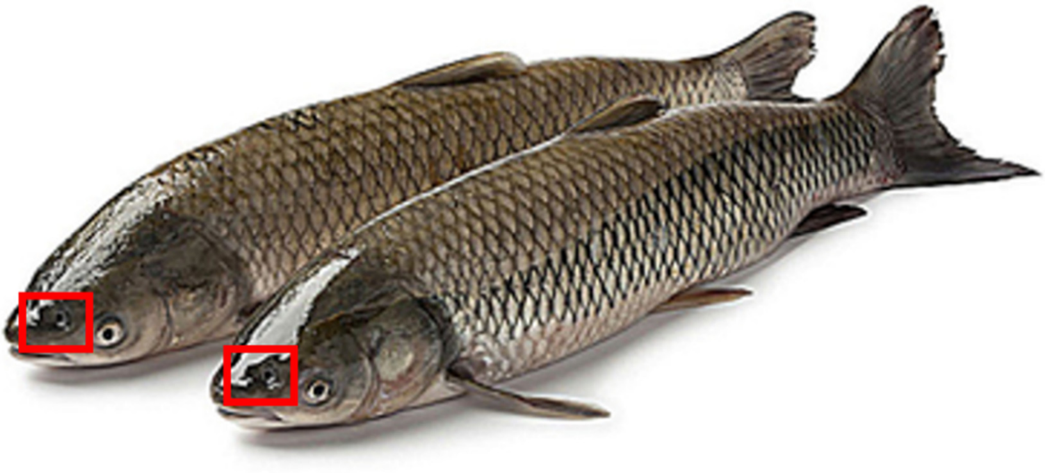

Figure S14. The appearance of adult grass carp. The area marked in the red box is the nasal cavity.
